# Sintilimab combined with chidamide in the treatment of extranodal nature killer/T-cell lymphoma with secondary hemophagocytic lymphohistiocytosis: Two case reports and literature review

**DOI:** 10.1097/MD.0000000000030731

**Published:** 2022-09-23

**Authors:** Qing-Yuan Xu, Hai-Yan Yang, Mei-Wei Li, Zhen-Dong He, Hao-Yuan Hong, Zhi-Gang Peng

**Affiliations:** a Department of Medical Oncology, First Affiliated Hospital of Guangxi Medical University, Nanning, Guangxi Zhuang Autonomous Region, P. R. China

**Keywords:** chidamide, extranodal nature killer/T-cell lymphoma, secondary hemophagocytic lymphohistiocytosis, sintilimab

## Abstract

Extranodal nature killer/T-cell lymphoma (ENKTL) failing in asparaginase-containing treatments is fatal, it has a higher mortality rate when accompanied by secondary hemophagocytic lymphohistiocytosis (HLH). The study reported 2 ENKTL-related HLH patients.

**Patient concerns:**

Patient 1 visited for nasal congestion and runny nose for 6 months then got a fever and serious myelosuppression after P-GEP (pegaspargase, gemcitabine, etoposide, and methylprednisolone) chemotherapy. Patient 2 complained of painless lymphadenectasis in the right neck for 4 months and experienced recurrent fever and poor performance status after 3 cycles of P-Gemox (pegaspargase, gemcitabine, and oxaliplatin) chemotherapy.

**Diagnoses:**

Patient 1 and patient 2 were diagnosed as ENKTL failing in asparaginase-based chemotherapy and involving secondary HLH.

**Interventions:**

The dose of chidamide was 20 mg twice a week for 2 weeks and sintilimab was 200 mg once every 3 weeks.

**Outcomes:**

ENKTL was relieved and the HLH was resolved after the therapy of sintilimab and chidamide. The patients had achieved durable survival without immune-related adverse events.

**Lessons:**

ENKTL-related HLH needs early diagnosis and treatment. The combined strategy of sintilimab plus chidamide help deal with HLH and solve ENKTL, it may be a useful treatment option for ENKTL-related HLH.

## 1. Introduction

Hemophagocytic lymphohistiocytosis (HLH) is characterized by inflammatory storms in over-immunization, divided into primary and secondary. Growing evidence suggests the pathogenesis of primary HLH are autosomal abnormalities or genetic defects, including gene mutation of PRF1, UNC13D, STX11, and STXBP2. Secondary HLH is frequently induced by infectious, autoimmune, and malignant tumor diseases, especially Epstein-Barr virus (EBV) or nature killer/T-cell lymphoma.^[1,2]^

HLH is life-threatening. The mortality rate of HLH is 20% to 88%, and malignant tumors related HLH patients have worse survival. Parikh et al reported 62 HLH patients, the median survival in tumor-induced and non-tumor patients was 1.4 months and 22.8 months, respectively.^[3]^ It is considered to use the standard treatments HLH-94 and HLH-2004 strategy primarily and the novel regimen of DEP (liposomal doxorubicin, etoposide, and methylprednisolone) for refractory HLH. However, A recent prospective study involving 63 eligible patients to receive salvage treatment in that DEP scheme after the failure of standard protocol showed an overall response rate was 76.2%, median survival was 28 weeks, 10 lymphoma-related EBV-positive patients still died of recurrent HLH caused by primary disease despite repeated DEP therapy.^[4]^ The features of HLH are recurrent and fatal, extranodal nature killer/T-cell lymphoma (ENKTL)-related HLH (ENKTL-HLH) is a fatal heterogeneous disease with no effective therapy. Therefore, the treatment of the original disease is very necessary to inhibit the recurrence of HLH and prolong the long-term survival.

In our case, this combination of sintilimab plus chidamide was a new strategy that did not contain chemotherapeutic drugs, which taking effect and safety in ENKTL-HLH patients.

## 2. Case report

### 2.1. Case1

A 36-year-old female went to another hospital with persistent nasal obstruction, runny nose, and nasal cavity perforation for 6 months, denying a history of hypertension, diabetes, heart disease, and infectious disease (Fig. 5A). No family history of cancer. The pathological, immunohistochemical examination and the computed tomography (CT) scan indicated ENKTL, stage II (imaging and pathological data could not be seen in another hospital). She underwent 3 cycles of P-GEP (pegaspargase, gemcitabine, etoposide, methylprednisolone) treatment. After 1 cycle of chemotherapy, grade IV myelosuppression occurred, and pegylated recombinant human granulocyte colony-stimulating factor (PEG-rhG-CSF) was given to improve leukocyte cells. However, her nasal soft tissue was more swollen than before, with a lot of pus in the nasal cavity at last (Fig. 5B). On April 20, 2020, she got a fever, the maximum body temperature was 40°.

**Figure 1.**
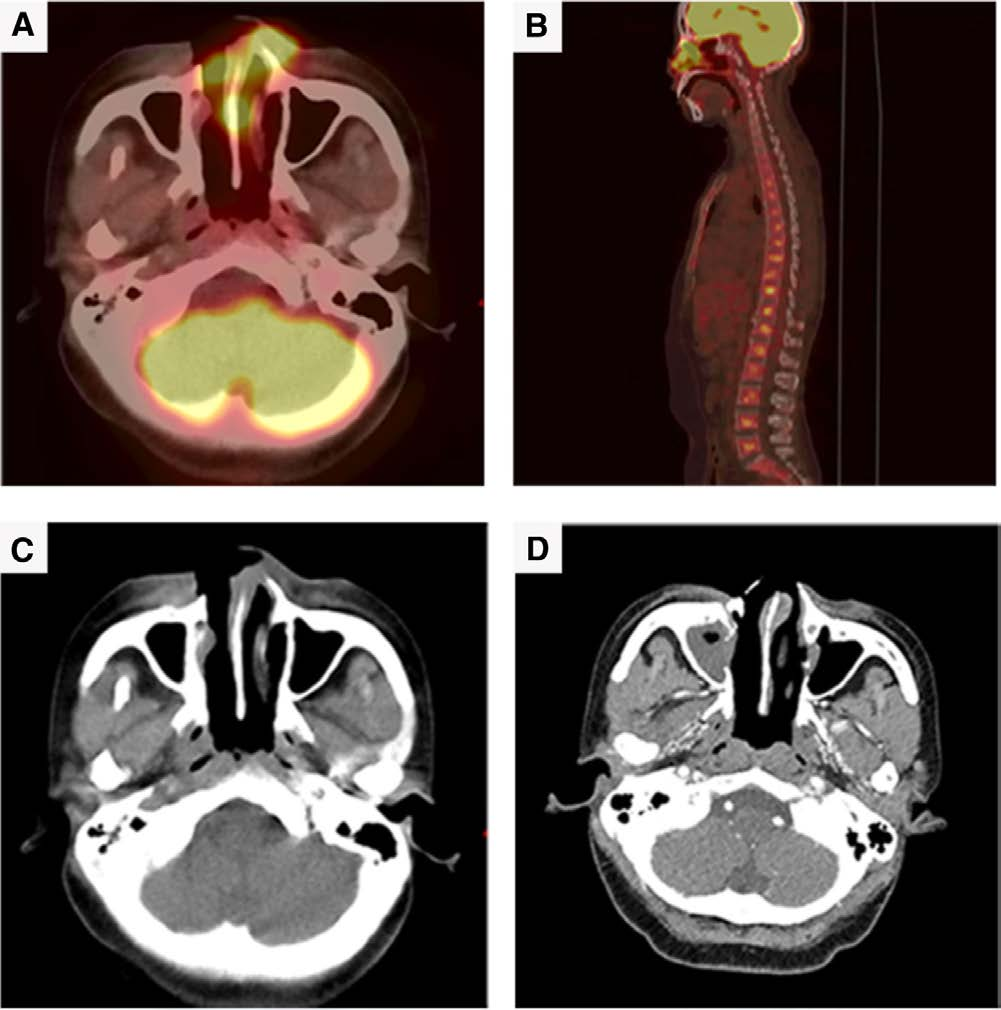
Images of case 1. (A) The maximum standardized glucose uptake value (SUVmax) of tissue in the right nasal, bilateral anterior and nasal septum was 4.8 on PET/CT scan after P-GEP treatment in May 2020. (B) The standardized glucose uptake value range was 2.0 to 4.8 in the spine in the sagittal section. (C) After the nasal biopsy, the thickened mass in the right nasal cavity, nasal septum and bilateral anterior nasal cavity on CT before immunotherapy. (D) After 2 cycles of immunotherapy, the swelling soft tissue was regressed in the bilateral nose adjacent to the face, the front part of the right nasal cavity and the septum subsided on enhanced CT in July 2020. PET/CT = positron emission tomography-computed tomography, SUVmax = the maximum standardized glucose uptake value.

**Figure 2.**
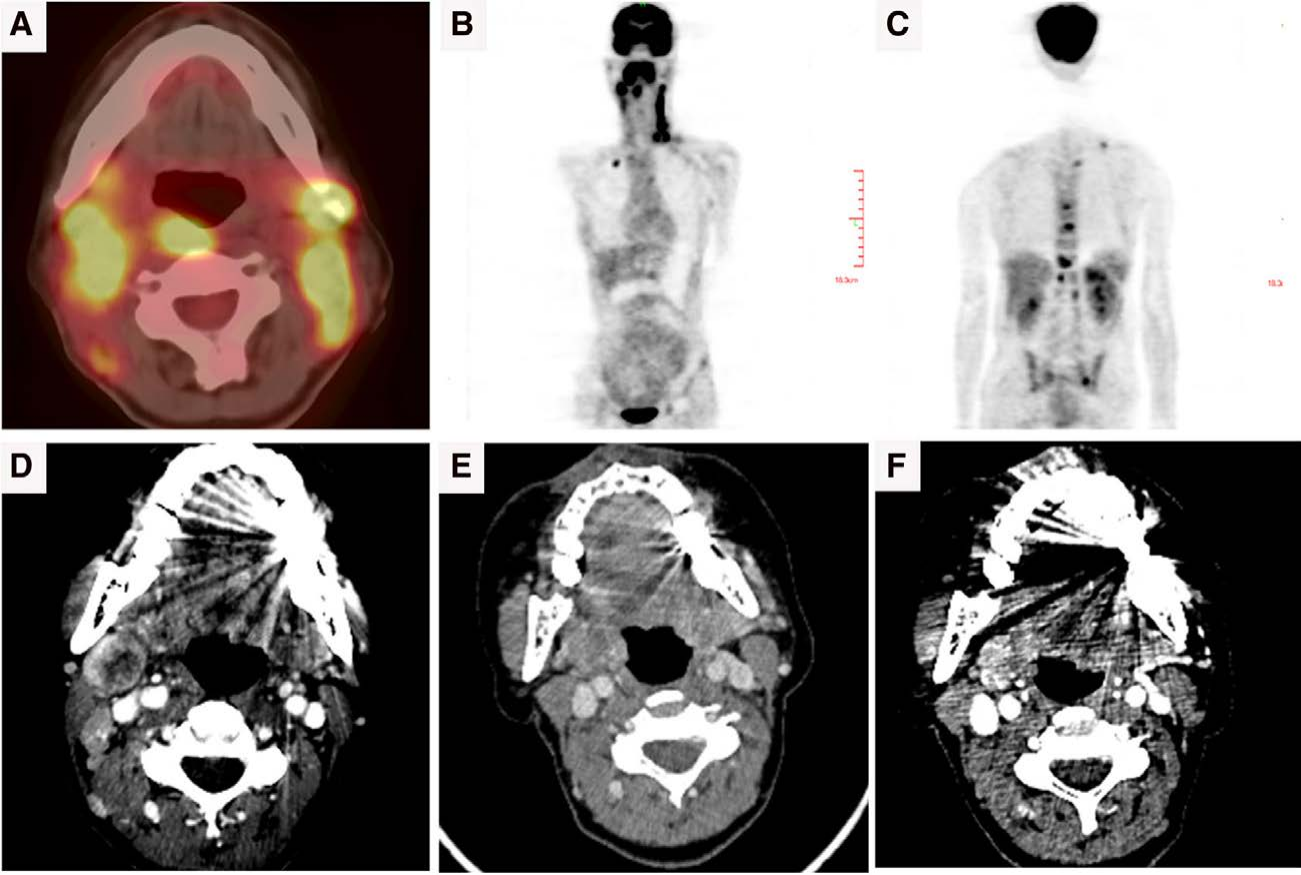
Images of case 2. (A)(B)(C) Significant 18F-FDG uptake in multiple sites on PET/CT scan, including soft tissues in the nasopharynx, right ethmoid sinus, soft palate, right oropharyngeal wall, and right tonsil, lymph nodes, bone and bone marrow, the maximum standardized glucose uptake value range from 6.9 to 8.3. Changes of the lymph node in the right neck on enhanced CT scan, including enlarged lymph node with necrosis in the first visit (about 2.5 cm × 2.2 cm) (D), stabled lymph node after P-Gemox chemotherapy (about 1.8 cm × 1.9 cm) (E), reduced lymph node (about 1.6 cm × 1.7 cm) after 5 cycles of immunotherapy (F). FDG = fluorodeoxyglucose, PET/CT = positron emission tomography-computed tomography.

**Figure 3.**
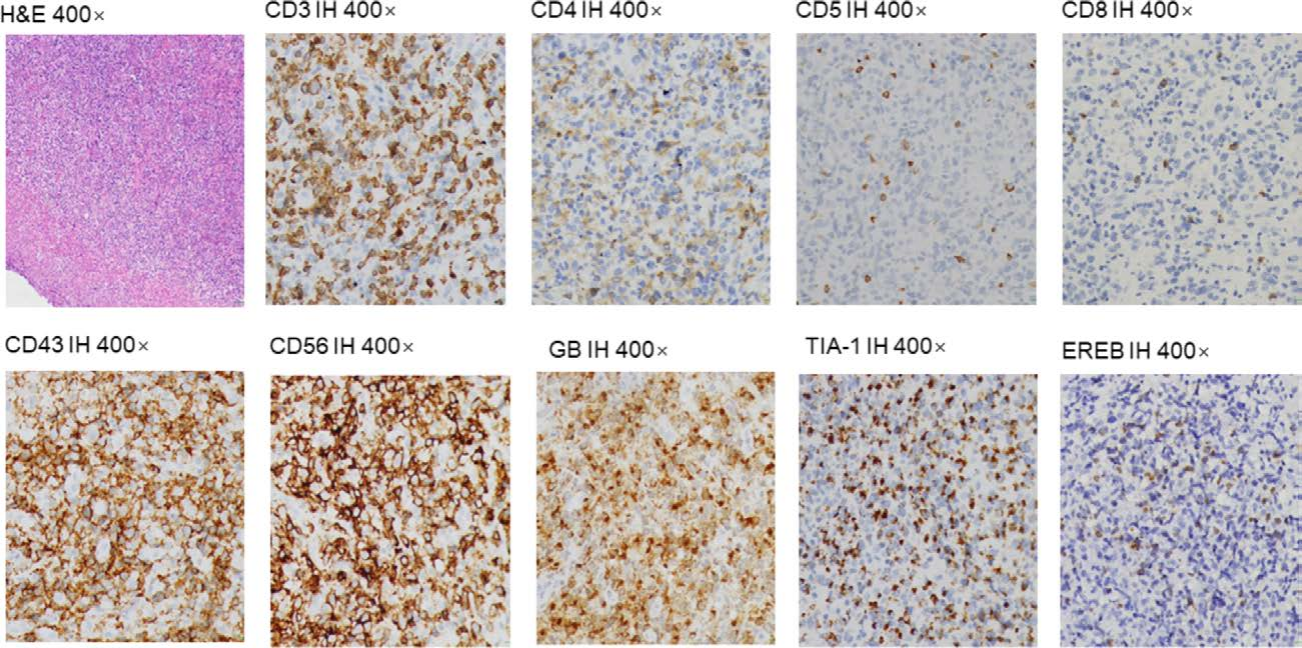
Pathologic analysis of case 1. H&E = hematoxylin and eosin, IH = immunohistochemical staining.

She was admitted to our hospital on May 8, 2020, for recurrent fever. The physical examination revealed the right nose was ulcerated and splenomegaly. We reexamined the nasopharyngeal mass. Microscopically, diffuse infiltration of medium-sized abnormal cells. Immunohistochemical staining of these heterosexual cells was positive with CD3, CD43, CD5, CD8, CD4, Bcl-6, CD56, Granzyme B (GB), and TlA-1, negative with CD21, CD20, CD15, Bcl-2, PAX-5, CD68, CD138, CD10, and MUM-1 (Fig. 3). Ki-67 protein was presented in 30% to 60% of lymphoma cells. In situ hybridization suggested EBV infected a few tumor cells. The [18F] fluorodeoxyglucose (FDG) positron emission tomography-computed tomography (PET/CT) scan showed tumor-active tissue in the right nasal cavity, the right ethmoid sinus, the maximum standardized glucose uptake value (SUV_max_) was 4.8, involved the anterior soft tissue of the bilateral nasal cavity, then nasal septum, and medial wall of the right maxillary sinus, the bone (SUV_max_ 2.0–4.8), and splenomegaly (SUV_max_ 2.0) (Fig. 1A, B, C). Considering to the clinical symptoms of recurrent fever and splenomegaly, HLH-related biochemical examinations were detected, the abnormal results were: hemoglobin: 63 g/L (reference range, 110–120 g/L), platelets: 89 × 10^9^/L (reference range, 100–300 × 10^9^/L), ferritin: 822.8 ng/ml (reference range, 0–500 ng/ml), low NK-cell activity of 15%, aspartate aminotransferase: 85 U/L (reference range, 10–40 U/L), lactate dehydrogenase: 363 U/L (reference range, 120–250 U/L), and EBV DNA was 4.96 × 10^4^ copy/ml (reference range, 0–400 copy/mL) (Fig. 6A). Bone marrow cytology presented hemophagocytosis. In addition, indexes of infectivity were: procalcitonin: 0.3 ng/mL (reference range, 0–0.05 ng/mL), C-reactive protein levels: 35.4 mg/L (reference range, 0–5 mg/L). Her disease worsened with secondary HLH. Subsequently, chidamide (20 mg, twice a week for 2 weeks) plus sintilimab (200 mg, once every 3 weeks) was employed on May 14, 2020, supplementing with anti-infective (cefodizime plus clindamycin) and nasal irrigation therapy. After 3 weeks, the biochemical indicators of HLH were normal, the value of EBV DNA down to 2.63 × 10^3^ copy/mL (Fig. 6A). After 2 cycles of immunotherapy, the clinical symptoms were relieved (Fig. 5C) and swelling tissue improved in the enhanced CT (Fig. 1D). The blood EBV descended to normal. The disease of ENKTL got a partial response (PR). She accepted another 3 cycles and did not have immune-related adverse events such as bone marrow suppression, hypothyroidism, or a lung infection. She was not uncomfortable from the last discharge to the previous follow-up time in June 2022 (Fig. 5D). The progression-free survival (PFS) was 24 months.

**Figure 4.**
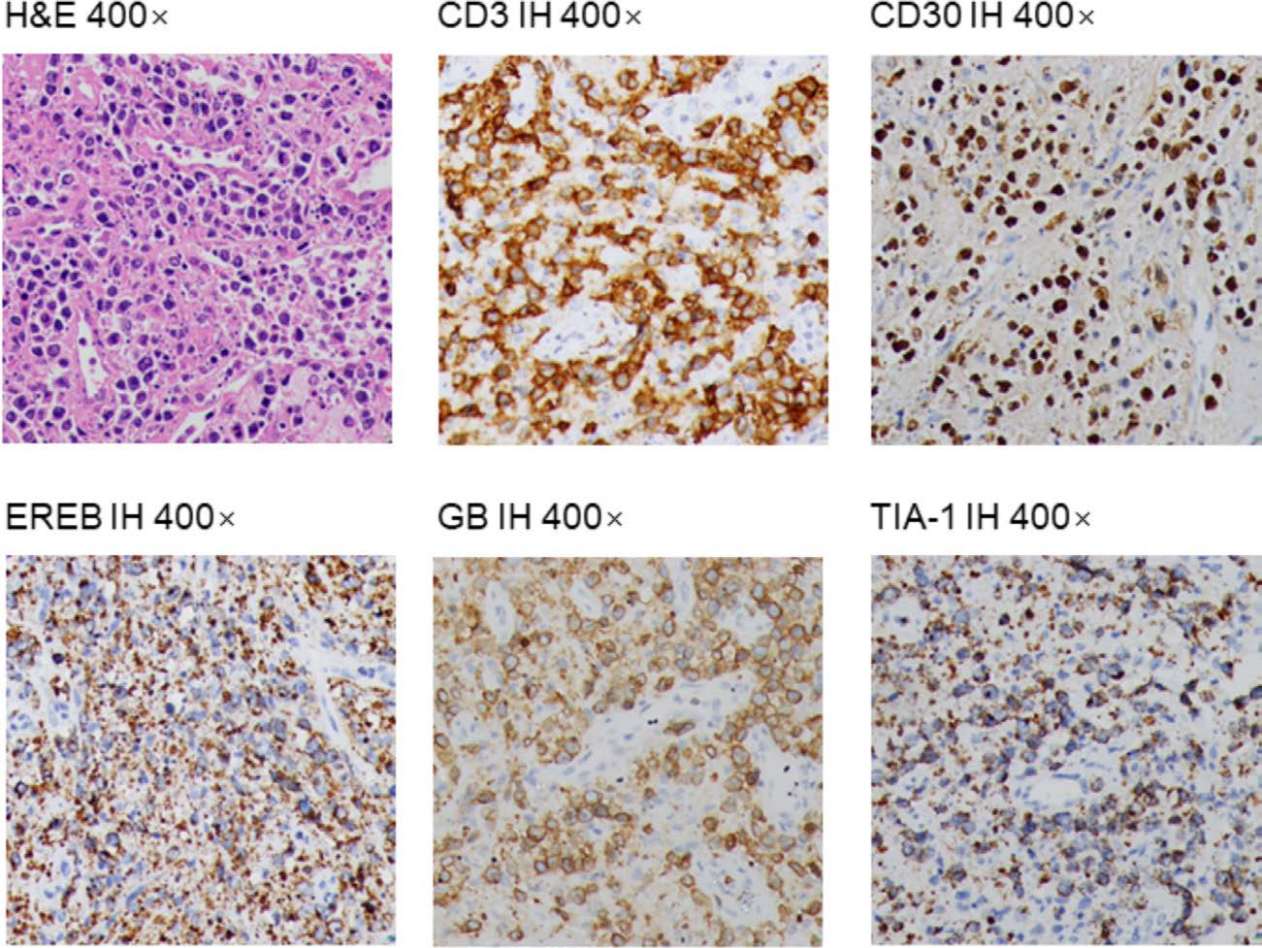
Pathologic analysis of case 2.

**Figure 5.**
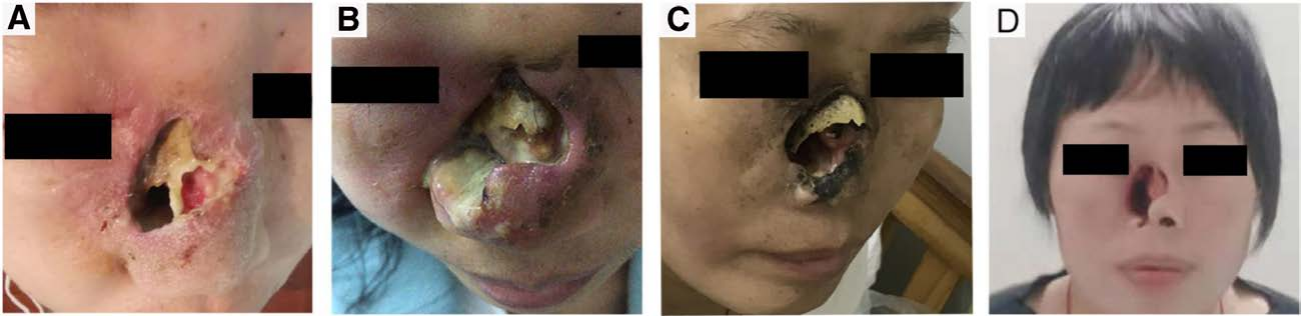
The nasal condition of case 1.

**Figure 6.**
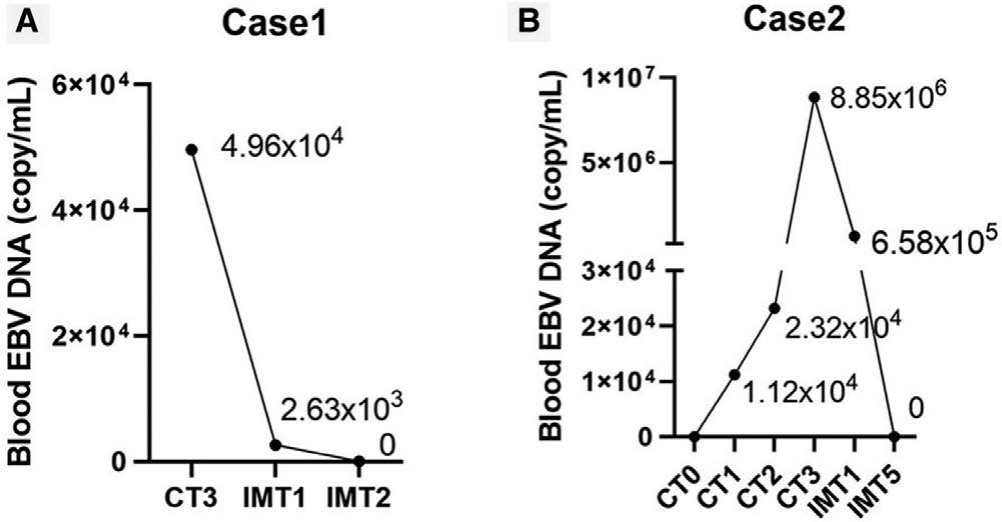
Changes of circulating EB virus in patients. CT = chemotherapy, EBV = Epstein-Barr virus, IMT = immunotherapy.

### 2.2. Case2

A 52-year-old female visited our hospital on July 22, 2020, due to painless lymphadenectasis in her right neck for 4 months, tinnitus and the lymph node larger for 10 days, did not have fever, cough or hoarseness. She had a history of right biliary calculus. No record of smoking, drinking, diabetes, hypertension or family history of cancer. We could touch a rugged and poorly mobile lymph node (the size was about a quail egg) behind the right ear. The albumin was 28.7 g/L in liver function. Blood routine, coagulation function, and renal function were regular. The enhanced CT scan showed metastatic lymph nodes in the bilateral neck, the size of the significant enlarged one was about 2.5 cm × 2.2 cm (Fig. 2D). The PET/CT scan showed increased glucose uptake in multiple soft tissues, including nasopharynx (SUV_max_ 8.3), right ethmoid sinus (SUV_max_ 8.3), soft palate (SUV_max_ 8.3), right oropharyngeal wall (SUV_max_ 8.3), and right tonsil (SUV_max_ 8.1), lymph nodes (SUV_max_ 6.9), bone and bone marrow (S SUV_max_ 6.1) (Fig. 2A, B, C). We took a biopsy from the lymph node. The pathological examination demonstrated a large area of necrosis in the lymph node and residual abnormal tumor cells scattered in the large piece of necrotic background. Immunohistochemistry was positive for CD3, CD30, Granzyme B, and TlA-1, while negative for CD56, CD4, CD8, CD43, CD5, CD20, PAX-5, MP0, and CK (Fig. 4). The ki-67 protein is expressed in 70% to 80% of lymphoma cells. Most tumor cells had EBV infection in situ hybridization. She was diagnosed with ENKTL, Ann Arbor stage IV.

Next, she accepted the first cycle of P-Gemox (pegaspargase, gemcitabine, oxaliplatin) treatment and became a fever. The inflammatory index increased: C-reactive protein was 71.1 mg/L (normal range, 0–5 mg/L) and procalcitonin was 0.203 ng/mL (normal range, 0–0.05 ng/mL). We considered the high body temperature was caused by nasal infection, not chemotherapy drugs. Thus, we continued the original chemotherapy strategy. Given anti-infective drugs sulperazon and vancomycin, the body temperature dropped normally. She got fever again and developed bone marrow suppression during the time of the second cycle of chemotherapy, but the diagnosis of HLH was inadequate. She had hyperpyrexia of 39.5°, asthenia, and poor physical condition after the third course of P-Gemox, without chest and abdominal pain. The EBV DNA increased sharply to 8.85 × 10^6^ copy/mL (Fig. 6B). The enhanced CT scan showed no effective remission (Fig. 2E). The disease developed to HLH, the indicators as follows were: hemoglobin 86 g/L (reference range, 110–120 g/L), platelets 18 × 10^9^/L (reference range, 100–300 × 10^9^/L), neutrophils 1.9 × 10^9^/L, low NK-cell activity of 5% (reference range, 9.5–23.5%), ferritin >40,000 ng/mL (reference range, 0–500 ng/mL), aspartate aminotransferase: 377 U/L (reference range, 10–40 U/L), lactate dehydrogenase: 2587 U/L (reference range, 120–250 U/L), and C-reactive protein levels: 79 mg/L (reference range, 0–5 mg/L), procalcitonin was 0.643 ng/mL (normal range, 0–0.05 ng/mL). The blood culture, (1,3)-β-D-glucan, and galactomannan were negative. Bone marrow examination revealed hemophagocytosis. On December 2, 2020, chidamide (20 mg, twice a week for 2 weeks) and sintilimab (200 mg, once every 3 weeks) were started; auxiliary therapies were liver protection and transfusion. Biochemical indicators of HLH fall to average after 4 weeks. After 5 cycles of immunotherapy, the circulating EBV decreased to undetectable (Fig. 6B), the right cervical lymph node was smaller (tumor size about 1.6 cm × 1.7 cm) (Fig. 2F) than before, SD was achieved. Up to the last follow-up time, the patient could work typically and did not occur adverse drug reaction events. The PFS was 17 months.

All procedures performed in this case involving human participants were in accordance with the Declaration of Helsinki (as revised in 2013). Written informed consent was obtained from the patient for publication of this case report and any accompanying images.

## 3. Discussion

ENKTL is a rare subtype of non-Hodgkin lymphoma. The pathogenesis and prognosis are closely related to the Epstein-Barr virus. The tumor mass most located in the nasal and upper aerodigestive tract, presents symptoms of organ involvement, such as nasal obstruction, nasal ulcer, hoarseness, etc, and belongs to a highly aggressive malignant tumor. HLH is a hyper-inflammatory response activated by macrophages often seen in advanced ENKTL, with has high interleukin-18 (IL-18) concentrations associated with the inflammatory storm.^[5,6]^ ENKTL-HLH has high mortality and lacks long-term follow-up, there is no standard treatment for it, so it is meaningful to explore the strategies of ENKTL-HLH.

HLH-94/HLH-2004 is still recommended as a standard scheme for HLH. However, ENKTL has heterogeneity, and the treatment of ENKTL-HLH is rarely reported in the literature. We found 9 specific regimens. The search conditions on PUBMED are as follow (“HPS” or “HLH” or “hemophagocytic syndromes” or “hemophagocytic lymphohistiocytosis”) and (“NK/T” or “ENKT” or “ENKTL” or “nature killer/T”). Including the regimens of DEP,^[7]^ L-asp-containing,^[8]^ ruxolitinib combined with doxorubicin, etoposide, and dexamethasone (R-DED),^[9]^ etoposide plus dexamethasone,^[10]^ pegaspargase-containing,^[11]^ dexamethasone and etoposide in combination with rituximab,^[12]^ dexamethasone, etoposide, ifosfamide, and carboplatin (DeVIC),^[13]^ methotrexate, etoposide, dexamethasone, pegaspargase (MEDA)^[14]^, and anti-programmed cell death 1 (PD-1) antibody.^[15]^ The studies recorded the shortest OS was 18 days and the longest was 916 days. According to the article of Wei et al, the independent prognosis factor of NK/T lymphoma patients was relapse/refractory ENKTL.^[10]^

Immune escape of tumor cells was associated with defective activation program of T cells in PD-1 and programmed cell death ligand 1 (PD-L1) signaling ways.^[16]^ The ENKTL cells high express PD-1 and PD-L1. Hence, using PD-1 and PD-L1 checkpoint inhibitors block the signal paths to perform an antitumor activity in ENKTL. Currently, the anti-PD-1 checkpoint inhibitors was proved to be effective in relapsed/refractory ENKTL with few adverse events, the medicine with good antitumor activity including pembrolizumab, nivolumab, sintilimab, and avelumab. Tao et al^[17]^ reported that 75% reached an objective response in 28 enrolled relapsed/refractory ENKTL patients using the single-drug of sintilimab, the treatment-related adverse events were grade 1 to 2. Therefore, PD-1 checkpoint inhibitors take effect in ENKTL and adverse drugs reaction is acceptable.

According to a few literature reports, PD-1 checkpoint inhibitors also play a role in HLH, the mechanism was correlated with the cytotoxic activation program of CD8 T cells normalization, and EBV cleared.^[18]^ Kwong et al reviewed 7 ENKTL cases reached a complete response, 5 patients experienced HLH among them, the symptoms and laboratory abnormalities resolved after 1 to 2 cycles of pembrolizumab treatment.^[15]^ A retrospective analysis involving 7 relapsed/refractory Epstein-Barr virus-related hemophagocytic lymphohistiocytosis (EBV-HLH) patients reported that 5 patients used nivolumab and remained in complete clinical remission.^[18]^ Sintilimab is a humanized anti-PD-1 antibody with a more significant affinity to bind to human PD-1 than nivolumab and pembrolizumab, it was cost-effective and acceptable. It has demonstrated activity in solid tumors, such as non-small cell lung cancer, liver cancer, and non-Hodgkin lymphoma. However, a multicenter phase 2 trial indicated a single drug of sintilimab had a low deep response rate in ENKTL^[17]^ which suggested that the combined treatments containing sintilimab may be promising options.

Chidamide is a novel histone deacetylase inhibitor, its effects include increasing the cytotoxicity of NK and CD8 + T cells, expand the expression level of PD-L1 to enhance immunotherapeutic response^[19]^ and regulating AKT/mTOR and MAPK pathways to inhibit the growth of NKTL cells.^[20]^ Zheng Yan et al documented pegaspargase-based chemotherapy and sintilimab-based immunotherapy fail in primary cutaneous NKTCL patients, until added the HDAC inhibitor received a complete molecular response.^[21]^ It has been validated that the combinations based on anti-PD-1 or PD-L1 have better antitumor efficacies and higher therapy response rates, including the addition of radiotherapy, chemotherapy, targeted therapy, etc.^[22]^ Combined the characteristic of PD-1 checkpoint inhibitor effective for ENKTL and HLH, but the low activity of the single drug, we try to add an immunomodulator chidamide for ENKTL-HLH, the result is effective at last. Multiple studies showed that sintilimab plus chidamide has a synergistic effect on the tumor, this therapy benefited advanced and metastatic sarcoma,^[23]^ peripheral T-cell lymphomas,^[24]^ and metastatic melanoma.^[25]^ Hence, we used this strategy for ENKTL, the outcome indicated it had a durable effect on ENKTL patients.

## 4. Conclusion

The case report showed the disease process of HLH and the clinical effects of sintilimab combined with chidamide. Uncontrollable ENKTL causes HLH, this combination could solve HLH and benefit primary disease, may be a promising treatment option for ENKTL-HLH patients, it is worth further investigation. Nevertheless, our data were from a small sample retrospective analysis, and we would collect more cases to verify.

## Author contributions

(I) Method design: Qing-Yuan Xu and Zhi-Gang Peng; (II) Data collection: Qing-Yuan Xu and Hai-Yan Yang; (III) Data analysis and interpretation: Qing-Yuan Xu, Hai-Yan Yang, Mei-Wei Li, Zhen-Dong He, and Hao-Yuan Hong; (IV) Manuscript writing: All authors; (V) Final approval of manuscript: all authors.

**Conceptualization:** Qing-Yuan Xu, Zhi-Gang Peng.

**Data curation:** Qing-Yuan Xu.

**Formal analysis:** Qing-Yuan Xu, Hai-Yan Yang, Mei-Wei Li, Hao-Yuan Hong.

**Writing – original draft:** Qing-Yuan Xu, Hai-Yan Yang, Zhi-Gang Peng, Mei-Wei Li, Zhen-Dong He, Hao-Yuan Hong.

**Writing – review & editing:** Hai-Yan Yang, Zhi-Gang Peng.
